# *Meloidogyne aberrans* sp. nov. (Nematoda: Meloidogynidae), a new root-knot nematode parasitizing kiwifruit in China

**DOI:** 10.1371/journal.pone.0182627

**Published:** 2017-08-30

**Authors:** Ye Tao, Chunling Xu, Chunfen Yuan, Honghong Wang, Borong Lin, Kan Zhuo, Jinling Liao

**Affiliations:** 1 Laboratory of Plant Nematology, South China Agricultural University, Guangzhou, China; 2 Guangdong Eco-Engineering Polytechnic, Guangzhou, China; Georg-August-Universitat Gottingen, GERMANY

## Abstract

High infection rates of roots of wild kiwifruit (*Actinidia chinensis* Planch) and soil infestation by a root-knot nematode were found in Anshun, GuiZhou Province, China. Morphology, esterase phenotype and molecular analyses confirmed that this nematode was different from previously described root-knot nematodes. In this report, the species is described, illustrated and named *Meloidogyne aberrans* sp. nov. The new species has a unique combination of characters. A prominent posterior protuberance, round and faint perineal pattern and a medium-length stylet (13.6–15.5 μm) characterized the females. Second-stage juveniles (J2) were characterized by a smooth lip region with distinctly protruded medial lips and a depression in outline at the oral aperture, a relatively long stylet (15.9–16.8 μm), four incisures in the lateral field and a very short, even poorly defined, hyaline tail terminus (2.2–5.5 μm). More incisures (11–15) existed in the lateral field of males, and the stylet and spicules of males were 18.2–19.6 μm and 22.7–36.8 μm long respectively. Egg masses were typically produced within the roots of kiwifruit. The new species had a rare Est phenotype, S2. Phylogenetic trees inferred from SSU, LSU D2D3, ITS, and partial *coxII*-16S rRNA revealed that *M*. *aberrans* sp. nov. was within the *Meloidogyne* clade and was distinguished from all described root-knot nematodes. Moreover, from histopathological observations, *M*. *aberrans* sp. nov. induced the formation of multinucleate giant cells.

## Introduction

The kiwifruit (*Actinidia chinensis* Planch), or Chinese gooseberry, is a favorite fruit that is eaten raw, made into juices or used as a garnish. Kiwifruit is currently grown in more than 20 countries, and in 2011, FAO estimated the area yielding kiwifruit reached 94,000 hm^2^ [1]. However, various diseases that include plant-parasitic nematodes threaten worldwide production of kiwifruit. *Meloidogyne* spp. root-knot nematodes are one of the most devastating plant pathogens to infest kiwifruit. With the exception of Africa, root-knot nematodes attack kiwifruit grown on other continents. *Meloidogyne incognita* and *M*. *hapla* are the most prevalent species to infect kiwifruit in the primary kiwifruit producing regions. The distribution of *M*. *incognita* includes Brazil, Chile, China, India, the United States and Turkey [2–7], and *M*. *hapla* occurs in Brazil, Chile, India, Italy, New Zealand, South Korea and Spain [5, 6, 8–12]. In addition to *M*. *incognita* and *M*. *hapla*, four other *Meloidogyne* species, *M*. *javanica*, *M*. *arenaria*, *M*. *ethiopica* and *M*. *actinidiae*, parasitize kiwifruit [6, 7, 13–18].

Kiwifruit is native to China, with areas of western provinces, including Shanxi, Sichuan and Guizhou Provinces, the primary kiwifruit planting districts. The primary kiwifruit production area in Guizhou Province is one of the important regions growing cultivated kiwifruit in China [1, 19–21]. In recent years, root-knot nematodes have developed into a serious problem in the primary kiwifruit production areas of Guizhou, with estimates that root-knot nematodes reduce yields of kiwifruit by 10–15%, and up to 40%, in some kiwifruit orchards of Guizhou [22]. A survey for *Meloidogyne* species in the damaged kiwifruit area was initiated in Guizhou Province, China. During this survey, one *Meloidogyne* population from kiwifruit that showed decline and low growth was found in Anshun, Guizhou Province. These root-knot nematodes, with females with an obvious posterior protuberance, were similar to nematodes that were once classified in the genus *Hypsoperine*; however, *Hypsoperine* has been synonymized with *Meloidogyne* [23]. Comparative morphological, morphometric, isozyme pattern and molecular studies of the nematode revealed differences with all other *Meloidogyne* species, particularly with those species once in the genus *Hypsoperine*. Therefore, the nematode is described as a new species, *Meloidogyne aberrans* sp. nov., in this report. Phylogenetic analyses based on small subunit (SSU), D2D3 expansion domains of large subunit (LSU D2D3), and internal transcribed spacer (ITS) rDNA sequences and one mitochondrial DNA (mtDNA) fragment located between the 3’ end of cytochrome oxidase subunit II (*coxII*) and the 5’ end of 16S rRNA (partial *coxII*-16S rRNA) were performed to investigate the relationship of *M*. *aberrans* sp. nov. with the DNA sequences available for other root-knot nematodes. Additionally, the host-parasite relationship was studied in naturally infected kiwifruit plants.

## Materials and methods

### Ethics statement

Specific permissions were not required for the nematodes collected for this study in Guizhou Province, China. The field used for nematode collection was neither privately owned nor protected and did not involve endangered or protected species.

### Nematode materials

Samples of kiwifruit roots and rhizosphere soils were collected in Anshun City, Guizhou Province, China, during February 2013, October 2015 and May 2017. Females, males and egg masses were dissected directly from galled roots. Second-stage juveniles (J2s) were isolated from fresh soils using Baermann funnels [24] or collected from hatching eggs [25].

### Morphological studies

To prepare for light microscopy (LM), males and J2s were relaxed with gentle heat, fixed in a solution of 4% formaldehyde + 1% glycerin and processed using the glycerin-ethanol method [24]. Perineal patterns of mature females were prepared as described [26]. The perineal pattern was trimmed and transferred to a drop of glycerin for observation. Nematodes were measured and photographed with a Nikon ECLIPSE Ni microscope equipped with a Nikon Digital Sight Camera and exclusive NIS-Elements BR software (Nikon, Tokyo, Japan).

Females, males and J2s were prepared for scanning electron microscopy (SEM) as described [27]. Nematodes were observed with a XL-30-ESEM microscope (Philips, the Netherlands).

### Isozyme phenotype analysis

Ten young, egg-laying females of *M*. *aberrans* sp. nov. were used for isozyme phenotype analysis. Four females of a previously identified population of *M*. *javanica* [28] were used for comparison. The phenotypes were for esterases (Est) and malate dehydrogenase (Mdh) [29].

### DNA extraction, amplification and sequencing

DNA was extracted from individual nematodes as described [30]. Three ribosomal DNA (rDNA) fragments (SSU, LSU D2D3 and ITS) and one mtDNA fragment (partial *coxII*-16S rRNA) of *M*. *aberrans* sp. nov. were amplified. SSU rDNA was amplified as two partially overlapping fragments as described [31]. For amplifying the two fragments, the primer pairs 988F (5’-CTCAAAGATTAAGCCATGC-3’)/1912R (5’-TTTACGGTCAGAACTAGGG-3’) [31] and 1813F (5’-CTGCGTGAGAGGTGAAAT-3’)/2646R (5’-GCTACCTTGTTACGACTTTT-3’) [31] were used. Primers for LSU D2D3 were D2A (5′-ACAAGTACCGTGAGGGAAAGTTG-3′) and D3B (5′-TCGGAAGGAACCAGCTACTA-3′) [32]. Primers for ITS were TW81 (5’-GTTTCCGTAGGTGAACCTGC-3’) and AB28 (5’- ATATGCTTAAGTTCAGCGGGT-3’) [33]. Primers for the mtDNA fragment were C2F3 (5′-GGTCAATGTTCAGAAATTTGTGG-3′) and 1108 (5′-TACCTTTGACCAATCACGCT-3′) [34]. Detailed protocols of PCR amplification for rDNA fragments and the mtDNA fragment were as described by Tanha Maafi *et al*. (2003) [35] and Powers and Harris (1993) [34], respectively. DNA sequencing was conducted as described [36]. The obtained sequences of SSU, LSU D2D3, ITS and partial *coxII*-16S rRNA were deposited in GenBank database.

### Phylogenetic analyses

The sequences of *M*. *aberrans* sp. nov. were compared with GenBank nematode sequences using the BLAST homology search program. The most similar sequences were selected for phylogenetic analyses. Out-group taxa for each data set were chosen according to previous molecular phylogenetic analyses for root-knot nematodes [37–39]. DNA sequences were aligned in MEGA4.0 [40] using default parameters. Models of base substitution were evaluated using MODELTEST3.7 [41, 42] combined with PAUP4.0 [43]. The Akaike-supported model, base frequencies, proportion of invariable sites, and gamma distribution shape parameters and substitution rates were used in phylogenetic analyses. Bayesian analysis was performed to confirm the tree topology for each gene separately using MrBayes 3.2 [42] running the chain for 1 × 10^6^ generations and setting the ‘burn-in’ at 2500. The MCMC (Markov Chain Monte Carlo) method was used within a Bayesian framework to estimate the posterior probabilities of the phylogenetic trees [44] and generate a 50% majority-rule consensus tree.

### Histopathology

Galled roots from kiwifruit plants naturally infected by *M*. *aberrans* sp. nov. were collected in Guizhou, China, for histopathological studies. Galls were cut off, fixed, dehydrated and embedded as described [45, 46]. Then, the galls were sliced, and the paraffin was removed following the description of Bachand and Castello (2001) [47]. Sections 10 μm thick were placed on glass slides, stained with safranin and fast green [48], mounted permanently in resinene, and examined and photographed with the Nikon ECLIPSE Ni microscope.

### Nomenclatural acts

The electronic edition of this article conformed to the requirements of the amended International Code of Zoological Nomenclature; therefore, the new names contained herein are available under that Code from the electronic edition of this article. This published work and the nomenclatural acts it contains have been registered in ZooBank, the online registration system for the ICZN. The ZooBank LSIDs (Life Science Identifiers) can be resolved and the associated information viewed through any standard web browser by appending the LSID to the prefix “http://zoobank.org/”. The LSID for this publication is as follows: urn:lsid:zoobank.org:pub: 75F0D6B5-58E5-4203-9669-30D3CC3C7B1C. The electronic edition of this work was published in a journal with an ISSN and has been archived and is available from the following digital repositories: PubMed Central, LOCKSS.

## Results

**Fig 1:**
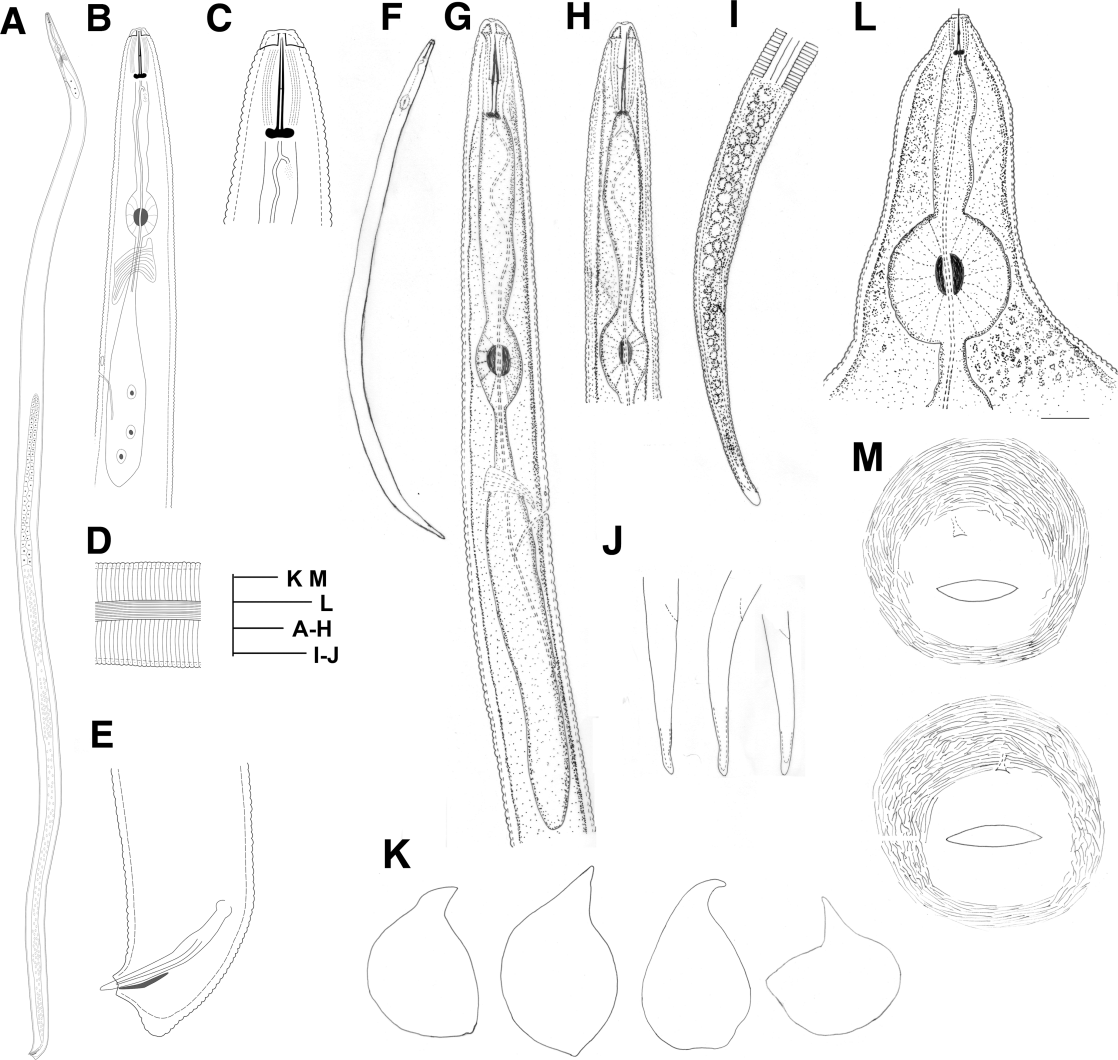
Line drawings of *Meloidogyne aberrans* sp. nov. (A) Entire body of male. (B) Pharyngeal region of male. (C) Head of male. (D) Lateral field of male. (E) Tail of male. (F) Entire body of J2. (G) Pharyngeal region of J2. (H) Anterior region of J2. (I) Lateral field and tail of J2. (J) Tail of J2. (K) Entire body of female. (L) Anterior region of female. (M) Perineal pattern. (Scale bars: A = 100 μm; B, D, E, M, I and J = 20 μm; C, G and H = 10 μm; F = 50 μm; K = 200 μm; L = 30 μm).

**Fig 2:**
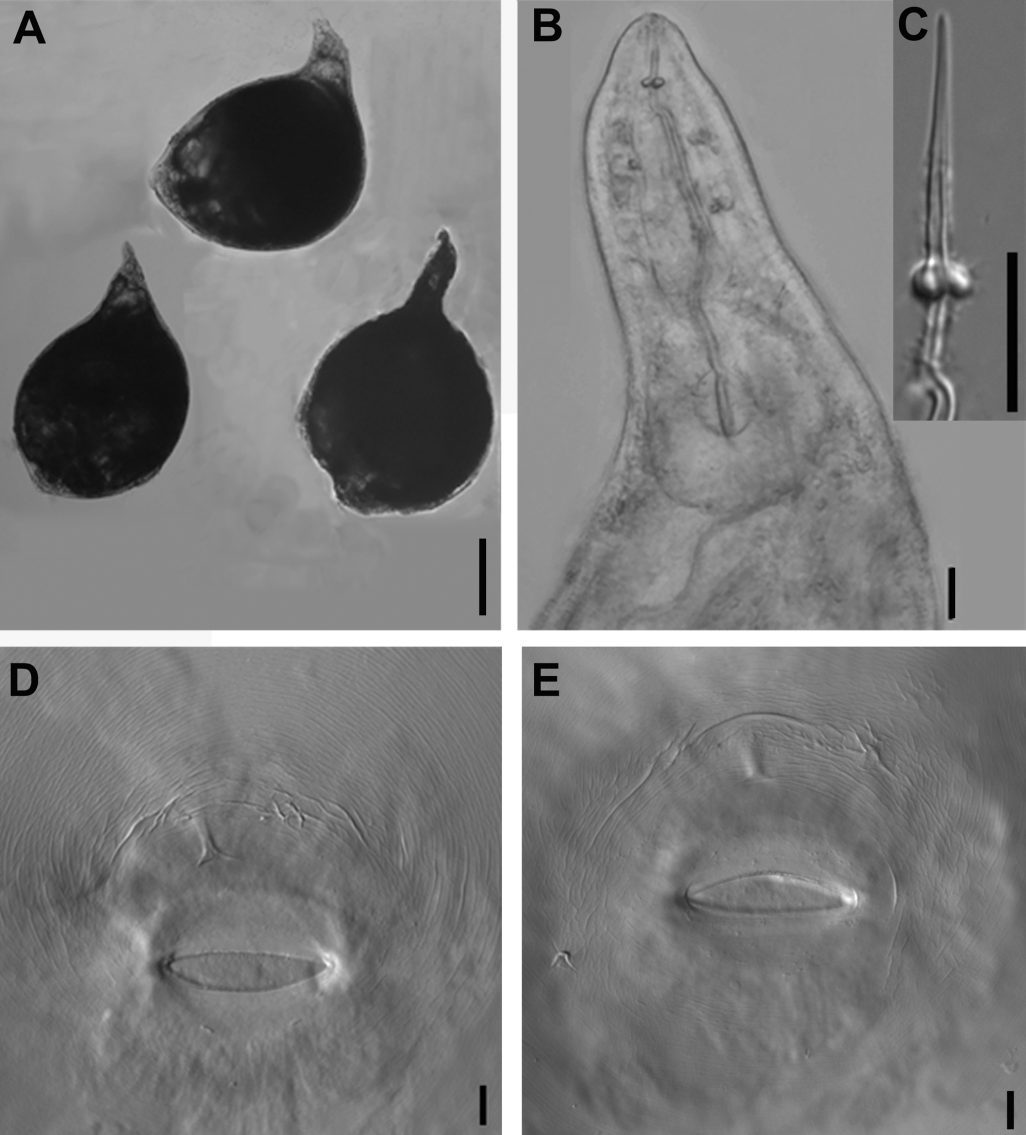
Photomicrographs of *Meloidogyne aberrans* sp. nov. females. (A) Entire body of female. (B) Anterior region of female. (C) Stylet of female. (D) and (E) Perineal pattern. (Scale bars: A = 200 μm; B-E = 10 μm).

**Fig 3:**
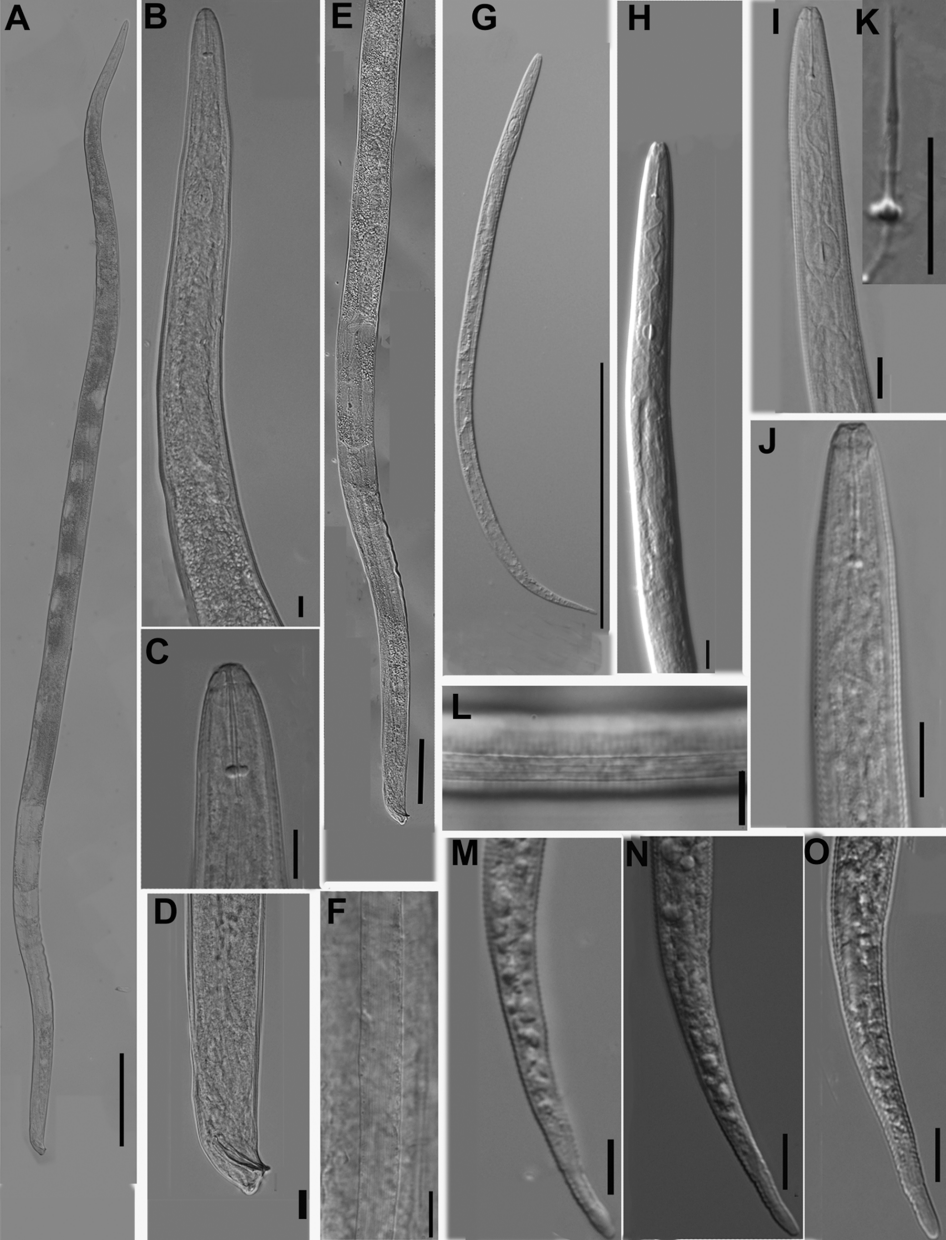
Photomicrographs of *Meloidogyne aberrans* sp. nov. males and J2s. (A) Entire body of male. (B) Pharyngeal region of male. (C) Anterior region of male. (D) Tail of male. (E) Posterior region and testis of male. (F) Lateral field of male. (G) Entire body of J2. (H) and (I) Pharyngeal region of J2. (J) Anterior region of J2. (K) Stylet of J2. (L) Lateral field of J2. (M), (N) and (O) Tail of J2. (Scale bars: A, G = 200 μm; B-D, F, H-O = 10 μm; E = 100 μm).

***Meloidogyne aberrans* sp. nov. Tao, Xu, Yuan, Wang, Lin, Zhuo & Liao sp. nov.** urn:lsid:zoobank.org:act:AC077264-94AB-4469-BBF0-E17DB07DB2F9 (Figs 1–3)

### Description

#### Female

**Fig 4:**
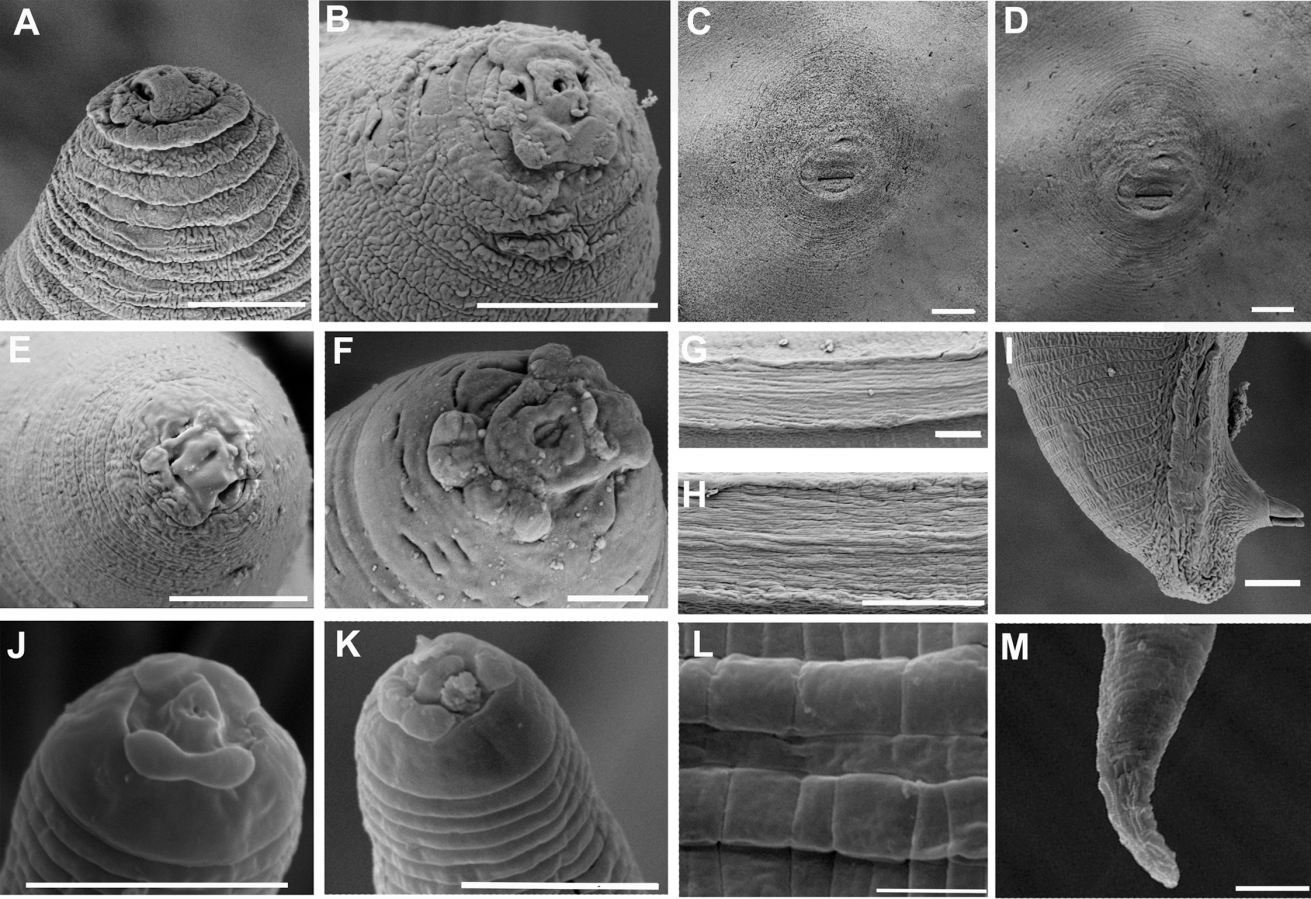
Scanning electron microscope photographs of *Meloidogyne aberrans* sp. nov. (A) and (B) Lip region of female in *en face* view. (C) and (D) Perineal pattern. (E) and (F) Lip region of male in *en face* view. (G) and (H) Lateral field of male. (I) Tail of male. (J) and (K) Lip region of J2 in *en face* view. (L) Lateral field of J2. (M) Tail of J2. (Scale bars: A, B, E-M = 5 μm; C, D = 20 μm).

Body completely embedded in galled tissue and pearly white, pear-shaped to ovoid with neck projecting at different angles. Posterior end of body with distinct, elevated perineum (Figs 1K and 2A). Lip region slightly offset. Head cap distinct, labial disk elevated (Figs 1L and 2B). Under SEM, the labial disc appeared round-squared, slightly elevated, fused with median lips, dumbbell-shaped. Six inner labial sensilla surrounding ovoid prestoma; stoma slit-like. Lateral lips large, triangular, separated from lip disc. Amphidial apertures elongated, located between labial disc and lateral lips (Fig 4A and 4B). Stylet moderately long, with round knobs, conus slightly curved and shaft straight (Fig 2C). Excretory pore distinct, typically located 2–3.5 stylet lengths posterior to stylet knobs. Metacorpus developed, rounded, with heavily sclerotized valve (Figs 1L and 2B). Pharyngeal gland with a large dorsal lobe and two subventral gland lobes. Perineal pattern oval, striae extremely faint, broken (Figs 1M, 2D and 2E). Vulva slit wider than vulva-anus distance. Anus fold visible in several specimens. Phasmid not visible. Measurements are listed in Table 1.

**Table 1:** Morphometrics of *Meloidogyne aberrans* sp. nov. All measurements are in μm and shown in the form: mean ± s.d. (range).

Character	Holotype Female	Paratype Females	Males	J2
n		25	10	27
L	884.5	938.1 ± 91.7 (806.2–1119.1)	1882.2 ± 162.7 (1701.5–2162.6)	451.7 ± 17.4 (419.2–473.8)
Maximum body diam.	544.7	581.6 ± 78.5 (441.3–712.6)	54.5 ± 3.7 (49.2–59.5)	14.7 ± 0.2 (14.4–15.2)
Neck length	306.4	282.8 ± 57.8 (184.4–378.0)	/	/
Stylet length	14.4	14.5 ± 0.6 (13.6–15.5)	18.9 ± 0.6 (18.2–19.6)	16.3 ± 0.3 (15.9–16.8)
DGO	4.8	4.5 ± 0.5 (3.7–5.8)	4.6 ± 0.6 (3.8–5.3)	3.3 ± 0.3 (3–3.9)
Metacorpus length	41.8	39.3 ± 3.7 (32.1–45.1)	18.2 ± 1.2 (16.7–19.9)	14.9 ± 1.2 (12.6–16.4)
Metacorpus width	40.6	38.4 ± 4.2 (31.5–46.6)	13.7 ± 1.8 (11.8–17)	8.2 ± 0.6 (7.4–9.3)
Metacorpus length/width	1.0	1.0 ± 0 (1–1.1)	1.3 ± 0.1 (1.2–1.4)	1.8 ± 0.2 (1.6–2.1)
Stylet knobs height	2.04	2.5 ± 0.3 (2–2.8)	2.5 ± 0.3 (2–2.9)	1.8 ± 0.2 (1.5–2.1)
Stylet knobs width	3.93	4.3 ± 0.3 (3.9–4.8)	4.7 ± 0.4 (4.1–5)	2.1 ± 0.1 (1.8–2.2)
Head region height	/	/	4.4 ± 1.1 (2.7–5.7)	2.4 ± 0.4 (1.6–2.8)
Head region width	/	/	9.2 ± 0.3 (8.8–9.5)	5 ± 0.4 (4.3–5.4)
Anterior end to excretory pore	50.4	48.1 ± 7.9 (32.0–57.8)	137.9 ± 5.1 (131.3–143.4)	86.7 ± 3.2 (80.5–91.6)
Anterior end to center of metacarpus	84.5	85.6 ± 13.7 (67.4–106.8)	78.9 ± 4.7 (73.4–86.3)	58 ± 2.6 (53.4–61.6)
Anterior end to cardia	/	/	/	106.1 ± 5.4 (94.3–113.5)
Anterior end to end of pharyngeal gland lobe	/	/	224.2 ± 18.5 (210.6–260.6)	191.8 ± 11.6 (175.2–210.1)
EP/ST	3.5	3.3 ± 0.6 (2.3–4.2)	/	/
Vulva length	31.8	33.6 ± 4.4 (23.7–41.1)	/	/
Distance from vulva to anus	23.3	23.1 ± 2.5 (17.8–27.1)	/	/
Anal body diameter	/	/	22.1 ± 2.6 (19.8–27.2)	9.4 ± 0.6 (8.2–10.4)
Tail length	/	/	9.4 ± 0.6 (8.8–10.2)	53 ± 2.6 (48.5–57)
Hyaline tail	/	/	/	3.6 ± 1.1 (2.2–5.5)
Spicules	/	/	31.5 ± 5 (22.7–36.8)	/
Gubermaculum	/	/	8.5 ± 0.8 (7–9.4)	/
a	1.6	1.6 ± 0.2 (1.3–2)	34.6 ± 2.7 (29.8–37)	30.7 ± 1.3 (28.9–32.7)
b	/	/	/	4.3 ± 0.3 (3.8–5)
b'	/	/	8.4 ± 1 (7.4–10.1)	2.4 ± 0.1 (2.2–2.5)
c	/	/	202.2 ± 27.4 (167.1–240.3)	8.5 ± 0.4 (7.9–9.3)
c'	/	/	0.4 ± 0.1 (0.3–0.5)	5.7 ± 0.4 (4.9–6.2)

#### Male

Body vermiform, tapering anteriorly (Figs 1A and 3A). Lip region slightly set off from body, with a obvious head cap (Figs 1C and 3C). Lip frame-work sclerotised. Under SEM, labial disc appeared round-squared, elevated. Large stoma-like slit located in a oval prestoma and surrounded by six inner labial sensilla. Medial lips large, separated from labial disc, forming an deep slit. Lateral lips large, triangular, separated from lip disc, with two or three interupting post-labial annulus. Amphidial apertures elongated, located between labial disc and lateral lips (Fig 4E and 4F). Stylet straight, cone narrow, sharply pointed; shaft widened slightly. Stylet knobs distinct, rounded and slightly concaved anteriorly (Fig 3C). Lateral fields narrow, occupying about one-fifth of the body width, with 11 to 15 lateral lines at mid-body, outer bands areolated in some specimens under SEM (Figs 1D, 3F, 4G and 4H). Excretory pore distinct, located posterior to nerve ring. Hemizonid conspicuous, located about 3–4 annuli anterior to excretory pore (Figs 1B and 3B). Metacorpus oval. One testis extending anteriorly (Fig 3E). Spicules of variable length, arcuate, slender, two pores clearly visible at tip under SEM (Figs 1E, 3D and 4I). Gubernaculum simple, almost straight (Figs 1E and 3D). Tail short, hemispherical, with a humped end and twisted posterior body portion (Figs 1E, 3D and 4I). Measurements are listed in Table 1.

#### J2

Body vermiform, tapering at both ends, ventrally curved after killing with heat (Figs 1F and 3G). Lip region smooth, continuous to body, depression in outline at oral aperture in the lateral view (Figs 1G, 1H and 3H–3J). Under SEM, labial disc appeared round-squared, and oral aperture located in the middle of labial disc surrounded by six inner labial sensilla. Medial lips distinctly protruded, extending farther than lateral lips and labial disc, resulting in an oral depression. Amphidial apertures appeared as a wide slit between the labial disc and lateral lips (Fig 4J and 4K). Stylet long, straight or conus slightly curved; cone narrow, sharply pointed; shaft widened slightly posteriorly; knobs distinct, sloping posteriorly (Fig 3K). Body annuli distinct, fine. Lateral fields with four lines (Figs 1I and 3L), areolated completely under SEM (Fig 4L). Excretory pore distinct, located posterior to nerve ring (Figs 1G and 3I). Hemizonid conspicuous, located 1–2 annuli anterior to excretory pore or immediately anterior to excretory pore. Metacorpus oval, with heavily sclerotized valve. Pharyngeal gland lobe long, ventrally overlapping intestine. Tail tapering gradually toward the end, with a bluntly round terminus (Figs 1I, 1J, 3M–3O and 4M). Hyaline tail short, sometimes not clearly defined (Figs 1I, 1J and 3M–3O). Phasmids indistinct. Measurements are listed in Table 1.

### Type host and locality

Roots and rhizosphere of kiwifruit (*Actinidia chinensis* Planch) were collected from Anshun City, Guizhou Province, China (26°13’ N, 106°13’ E).

### Etymology

The species epithet refers to the unique combination of morphological characters, which included an elevated perineum, a faint perineal pattern, distinctly protruded medial lips resulting in a depression in outline at the J2 oral aperture and a very short, even poorly defined hyaline tail.

### Type material

Holotype female. Female perineal patterns and paratype males and J2s are deposited in the nematode collection of the author at the Laboratory of Plant Nematology, South China Agricultural University, Guangzhou, China. Additional female and J2 paratypes are distributed in the USDA Nematode Collection, Beltsville, Maryland, USA, and the Canadian National Nematode Collection, Ottawa, Canada.

### Diagnosis and relationships

*Meloidogyne aberrans* sp. nov. has a unique combination of characters. A prominent posterior protuberance, round and faint perineal pattern and a medium-length stylet (13.6–15.5 μm) characterized females. Males with stylet 18.2–19.6 μm long, spicules 22.7–36.8 μm long and 11–15 lateral lines. J2s were characterized by a smooth lip region with distinct protruded medial lips and a depression in outline at the oral aperture, a relatively long stylet (15.9–16.8 μm), four incisures in the lateral field and a very short, even not clearly defined, hyaline tail terminus (2.2–5.5 μm). And *M*. *aberrans* sp. nov has specific SSU, LSU D2-D3, ITS and partial *coxII-16S* rRNA sequences.

Because of the prominent posterior protuberance in females, *M*. *aberrans* sp. nov. is similar to those species that have an elevated perineum, including *M*. *ichinohei* Araki, 1992 [49]; *M*. *acronea* Coetzee, 1956 [50]; *M*. *africana* Whitehead, 1959 [51,52]; *M*. *graminis* (Sledge and Golden, 1964) Whitehead, 1968 [53,54]; *M*. *megadora* Whitehead, 1968 [52,54,55]; *M*. *mersa* Siddiqi & Booth, 1991 [56]; *M*. *ottersoni* (Thorne, 1969) Franklin, 1971 [57,58]; *M*. *propora* Spaull, 1977 [59]; *M*. *spartinae* (Rau and Fassuliotis, 1965) Whitehead, 1968 [54,60]; *M*. *oryzae* Maas, 1978 [61]; *M*. *salasi* Lopez, 1984 [62] and *M*. *triticoryzae* Gaur, 1993 [63]. First, the new species was easily distinguished from these twelve species by a depression in outline at the oral aperture. Then, the new species differed from *M*. *ichinohei* by the longer female, male and J2 stylet (13.6–15.5 vs. 11.0–13.6 μm; 18.2–19.6 vs. 16.6–17.4 μm; 15.9–16.8 vs. 9.7–12.9 μm), the larger male length (1701.5–2162.6 vs. 1450.8–1581.0 μm), the lower DGO of male (3.8–5.3 vs. 6.1–6.9 μm), the shorter male tail (8.8–10.2 vs. 12.8–13.8 μm), more lateral lines in males (11–15 vs. 7–8) and fewer incisures in the J2 lateral field (4 vs. 6); from *M*. *acronea* by the longer female, male and J2 stylet (13.6–15.5 vs. 10.0–14 μm; 18.2–19.6 vs. 16–18 μm; 15.9–16.8 vs. 9.7–12 μm), more lateral lines in males (11–15 vs. 4), the shorter male tail (c = 167.1–240.3 vs. 138–150) and the male tail shape (humped tail terminus vs. blunt tail tip); from *M*. *africana* by the larger female (806.2–1119.1 vs. 400–770 μm in body length; 441.3–712.6 vs. 300–540 in maximum body width), the longer male and J2 sytlet (18.2–19.6 vs. 14.0–18.0 μm; 15.9–16.8 vs. 10.5–12.5 μm), male body (1701.5–2162.6 vs. 816–1750 μm) and J2 tail (48.5–57 vs. 39.0–46.0 μm; c’ = 4.9–6.2 vs. 3.5–4.7), the shorter hyaline tail (2.2–5.5 vs. 8.0–13.0 μm) and the different male tail (humped tail terminus vs. round tail ternimus); from *M*. *graminis* by the different perineal pattern (oval, extremely faint, without incisures vs. coarse, with an incisure and a high arch), the longer female and J2 stylet (13.6–15.5 vs. 11.7–13.44 μm; 15.9–16.8 vs. 11.7–13.44 μm), more posterior excretory pore position in females (2–3.5 stylet lengths posterior to stylet knobs vs. level with stylet knobs), the shorter J2 tail (48.5–57 vs. 68–88 μm; c = 7.9–9.3 vs. 5.7–6.78) and hyaline tail (2.2–5.5 vs. 14.0–22.4 μm), the lower ratio a of males (29.8–37 vs. 37.38–50.39) and more lateral lines in males (11–15 vs. 4); from *M*. *megadora* by the longer J2 stylet (15.9–16.8 vs. 10.7–13.2 μm), the different J2 tail (blunt rounded terminus vs. tail tapering irregularly ending in a subacute variously shaped end), the shorter hyaline tail (2.2–5.5 vs. 8.0–23.0 μm), the higher ratio b of J2 (3.5–5 vs. 2.08–3.00 μm), the lower ratio a of male (29.8–37 vs. 36.9–62.8 μm) and more incisures in the male lateral field (11–15 vs. 4–6); from *M*. *mersa* by the shorter male stylet (18.2–19.6 vs. 20–23 μm), female and J2 body (806.2–1119.1 vs. 1150–2530 μm; 419.2–473.8 vs. 610–870 μm), J2 tail (48.5–57 vs. 63.8–81 μm; c’ = 4.9–6.2 vs. 5.7–10.5), hyaline tail (2.2–5.5 vs. 8–13 μm), spicules (22.7–36.8 vs. 35–39 μm) and gubermaculum (7–9.4 vs. 10–16 μm), the lower ratio a of female, male and J2 (1.3–2 vs. 1.8–4.5; 29.8–37 vs. 40–66; 28.9–32.7 vs. 39–58), the longer J2 stylet (15.9–16.8 vs. 13.0–16.0 μm) and more lateral lines in males (11–15 vs. 6); from *M*. *ottersoni* by the larger female (806.2–1119.1 vs. 390–520 μm in length; 441.3–712.6 vs. 180–320 μm in diameter), the longer female, male and J2 stylet (13.6–15.5 vs. 10–12 μm; 18.2–19.6 vs. 14–16 μm; 15.9–16.8 vs. 13–15 μm), spicules (22.7–36.8 vs. 19–23 μm) and gubermaculum (7–9.4 vs. 3–4 μm), more posterior excretory pore position in females (2–3.5 stylet lengths posterior to stylet knobs vs. almost opposite stylet knobs), the tail terminus shape of J2 (bluntly round vs. irregularly clavate or knobbed) and more lateral lines in males (11–15 vs. 4); from *M*. *propora* by the perineal pattern (without incisures vs. with a single, broken and weak incisure), more posterior excretory pore position in females (32.0–57.8 vs. 19–30 μm, from anterior end to excretory pore), the smaller distance from vulva to anus (17.8–27.1 vs. 32–53 μm), fewer J2 lip annuli (0 vs. 1), the shorter J2 stylet (15.9–16.8 vs. 16.5–18.5 μm), the higher ratio a of J2 (28.9–32.7 vs. 17–25) and ratio c of male (167.1–240.3 vs. 80–149), the lower ratio a of male (29.8–37 vs. 36–49 μm), the longer J2 tail (48.5–57 vs. 15.9–21.7 μm; c = 7.9–9.3 vs. 17.3–24.1; c’ = 4.9–6.2 vs. 1.3–1.8) and more lateral lines in males (11–15 vs. 6 or 7); from *M*. *spartinae* by the tail terminus shape of J2 (bluntly round vs. spiked and bulbous), more posterior excretory pore position in females (32.0–57.8 vs. 19–30 μm, from anterior end to excretory pore), more incisures in the J2 lateral field (4 vs. 3), the lower ratio a of J2 (28.9–32.7 vs. 43.2–65.1), the shorter J2 (419.2–473.8 vs. 612–912 μm), J2 tail (48.5–57 vs. 77–113.4 μm) and hyaline tail (2.2–5.5 vs. 16.8–28 μm); From *M*. *oryzae* by the larger female (806.2–1119.1 vs. 475–750 μm in length; 441.3–712.6 vs. 250–432 μm in maximum body width), the longer female neck (184.4–378.0 vs. 80–136 μm) and J2 stylet (15.9–16.8 vs. 14–15 μm), the lower female DGO (3.7–5.8 vs. 7 μm), ratio b’ of J2 (2.2–2.5 vs. 7.2–9.8), ratio c’ of J2 (4.9–6.2 vs. 6.8–9.0 μm) and ratio a of male (29.8–37 vs. 44–68 μm), the shorter J2 (419.2–473.8 vs. 500–615 μm), J2 tail (48.5–57.0 vs. 70–90 μm) and hyaline tail (2.2–5.5 vs. 14–26 μm) and more lateral lines in males (11–15 vs. 3–7); from *M*. *salasi* by the larger female (806.2–1119.1 vs. 372.0–625.0 μm in body length; 441.3–712.6 vs. 209.0–425.0 μm in maximum body width), the longer female and J2 stylet (13.6–15.5 vs. 8.1–12.5 μm; 15.9–16.8 vs. 9.2–13.3 μm), the smaller maximum body width of J2 (14.4–15.2 vs. 15.3–19.3 μm), the higher ratio c of J2 (7.9–9.3 vs. 5.9–7.7 μm) and more lateral lines in males (11–15 vs. 4); from *M*. *triticoryzae* by the larger female (806.2–1119.1 vs. 330–480 μm in length; 441.3–712.6 vs. 200–320 μm in maximum body width), the longer female neck (184.4–378.0 vs. 110–185 μm) and J2 stylet (15.9–16.8 vs. 11.5–13.0 μm), the higher DGO of female (3.7–5.8 vs. 2–4 μm), ratio EP/ST of female (2.3–4.2 vs. 1.4–1.6 μm) and ratio c of J2 (7.9–9.3 vs. 5.7–7.4), the bigger metacarpus of female (32.1–45.1 × 31.5–46.6 vs. 24–28 × 21–23 μm), more posterior excretory pore postition in J2s (86.7–91.6 vs. 63–68 μm from anterior end to excretory pore), the lower ratio b’ of J2 (2.2–2.5 vs. 3.4–4.4), the shorter hyaline tail (2.2–5.5 vs. 16–19 μm) and more lateral lines in males (11–15 vs. 4).

Compared with the other six species reported from kiwifruit, including *M*. *actinidiae* Li and Yu, 1991, *M*. *ethiopica* Whitehead, 1968 and four common species, *M*. *arenaria* (Neal, 1889) [64] Chitwood, 1949 [65], *M*. *hapla* Chitwood, 1949 [65], *M*. *incognita* (Kofoid & White, 1919) [66] Chitwood, 1949 [65] and *M*. *javanica* (Treub, 1885) Chitwood, 1949 [65,67], the new species was easily distinguished by the elevated perineum, faint perineal pattern, depression in outline at the J2 oral aperture, longer J2 stylet (15.9–16.8 vs. < 15 μm) and J2 tail terminus shape (bluntly rounded vs. subacute or fine rounded).

### Isozyme analysis

**Fig 5:**
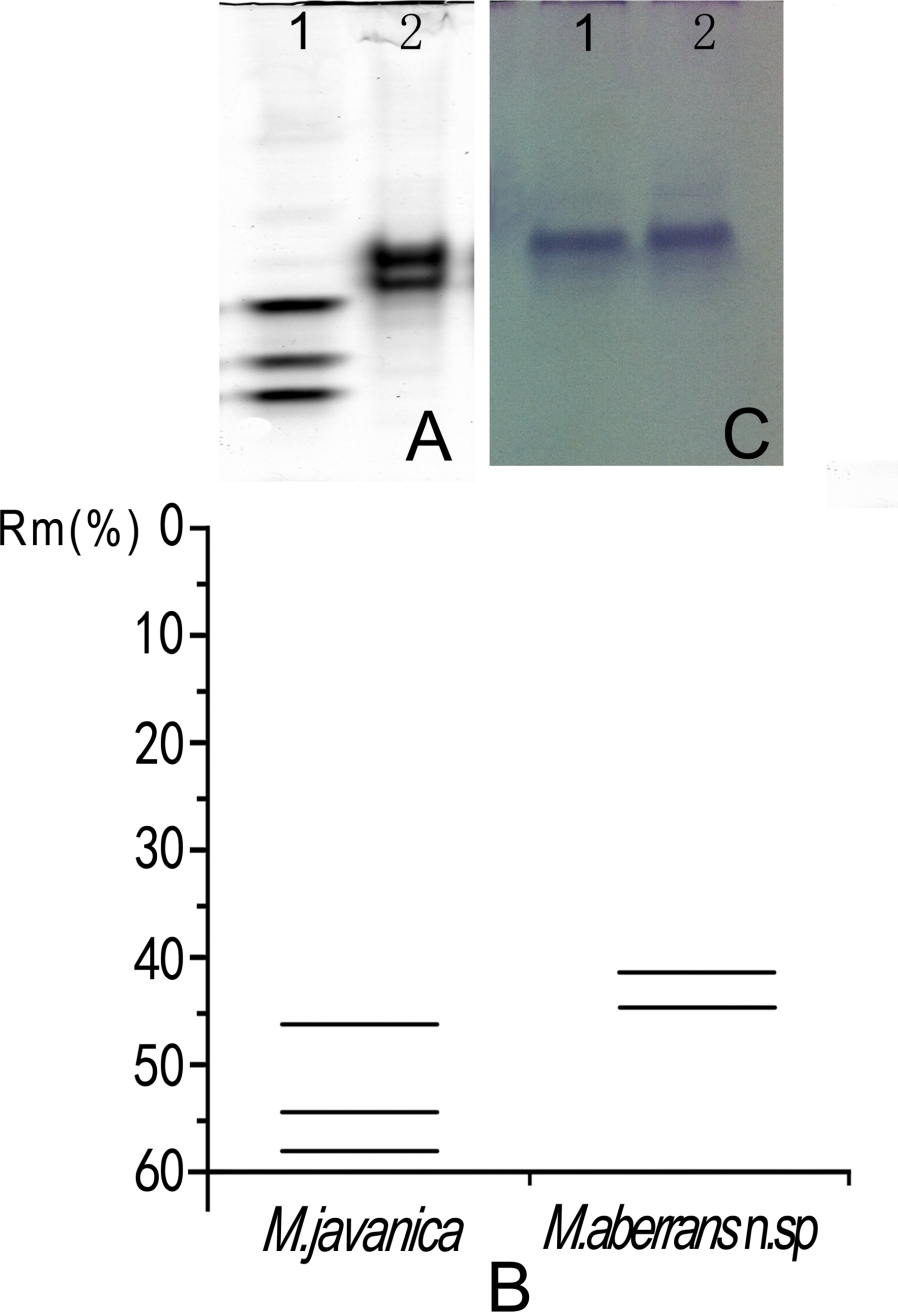
Esterase and malate dehydrogenase phenotype electrophoresis patterns of protein homogenates from ten young, egg-laying females of *Meloidogyne aberrans* sp. nov. (lane 2) and from four young, egg-laying females of *M*. *javanica* as a reference population (lane 1). (A) Esterase patterns. (B) Relative mobility (Rm) of esterase bands. (C) Malate dehydrogenase patterns.

The isozyme electrophoretic analysis of young, egg-laying females of *M*. *aberrans* sp. nov. showed a rare Est phenotype, S2, i.e., two Est bands at Rm = 40.5% and 44.5% (Fig 5A and 5B). The band of Mdh phenotype of *M*. *aberrans* sp. nov. was similar in size to that of *M*. *javanica* N1 Mdh phenotype (Fig 5C).

### Molecular characterization

The five SSU sequences of 1734 bp from one female, one male and three different J2s were sequenced, respectively. GenBank accession numbers of the five sequnences are KF278755 for the female, KF278756 for the male, and KX776409, KX776410 and KU598836 for the J2s. The identities were 100% or 99.9% (1733/1734) between any two of the five. A BLAST search of *M*. *aberrans* n. sp. revealed the highest match with the sequence of *M*. *ichinohei* (GenBank accession numbers EU669953). The identities between the five sequences from the new species and the sequence from *M*. *ichinohei* were 93.7%.

Four LSU D2D3 sequences of 789 bp and one J2 LSU D2D3 sequence of 791 bp were sequenced based on the same templates as mentioned above. GenBank accession numbers are KF278754 for the female, KF278753 for the male, and KX776411, KX776412 and KU598837 for the J2s. The identities of these five sequences were 100% or 99.7% (787/791) with two insertions/deletions between any two. A BLAST search of *M*. *aberrans* n. sp. revealed the highest match with the sequence of *M*. *ichinohei* (GenBank accession numbers EF029862). However, the identities between the five sequences from the new species and the sequence from *M*. *ichinohei* were only 83.5%.

The five ITS-rDNA sequences of 664 bp were sequenced based on the same templates as mentioned above. GenBank accession numbers of these sequences are KF278757 for the female, KF278758 for the male, and KX776413, KX776414 and KU598838 for the J2s. The identities were 100%, 99.8% (663/664) or 99.7% (662/664) between any two of the five. A BLAST search of *M*. *aberrans* n. sp. revealed the highest match with the sequence of *M*. *panyuensis* (GenBank accession numbers AY394719). The identities between the sequences from the new species and the sequence from *M*. *panyuensis* were only 77.4% and 77.3%, respectively.

The three sequences of 549 bp, one male sequence of 548 bp and one J2 sequence of 547 bp for *coxII-*16S rRNA were sequenced based on the same templates as mentioned above. GenBank accession numbers are KF278759 for the female, KF278760 for the male, and KX776415, KX776416 and KU598839 for the J2s. Among these five sequences, the identities were 100%, 99.8% (548/549) or 99.6% (547/549 or 546/549 with one insertions/deletions) between any two. A BLAST search of *M*. *aberrans* n. sp. revealed the highest match with the sequenc of *M*. *marylandi* (GenBank accession numbers KC473862). The identities between the sequences from the new species and the sequence from *M*. *marylandi* were only 76.5%-76.7%.

These twenty different sequences, including rDNA sequences of SSU, LSU D2D3 and ITS, and mtDNA sequence of *coxII-*16S rRNA, of *M*. *aberrans* sp. nov., indicated that all had high-scoring matches with some *Meloidogyne* species and that all were clearly different from those in the GenBank database. Sequence divergences between the new species and other species of *Meloidogyne* were 5.4–11.1%, 18.8–33.7%, 26.5–67.0% and 22.9–38.0% for SSU, LSU D2D3, ITS and *coxII-*16S rRNA, respectively, supporting its separate specific status.

**Fig 6:**
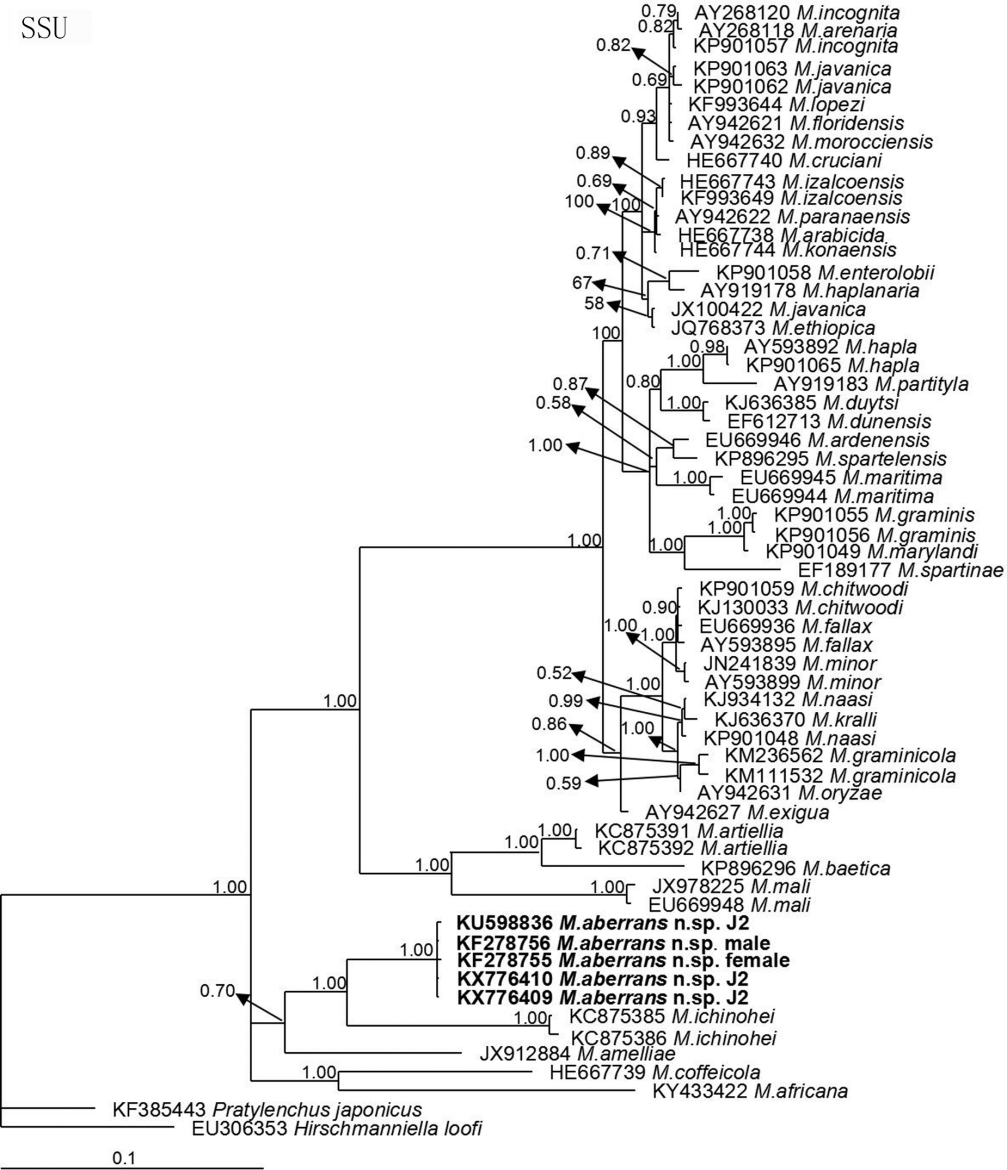
Bayesian consensus tree inferred from SSU of *Meloidogyne aberrans* sp. nov. under GTR+I+G (lnL = 8867.3428; AIC = 17754.6855; freqA = 0.2548; freqC = 0.2147; freqG = 0.2721; freqT = 0.2584; R(a) = 1.3844; R(b) = 3.0808; R(c) = 2.0934; R(d) = 0.6800; R(e) = 5.8377; R(f) = 1; Pinva = 0.5046; Shape = 0.6289). Posterior probability values exceeding 50% are given for appropriate clades.

**Fig 7:**
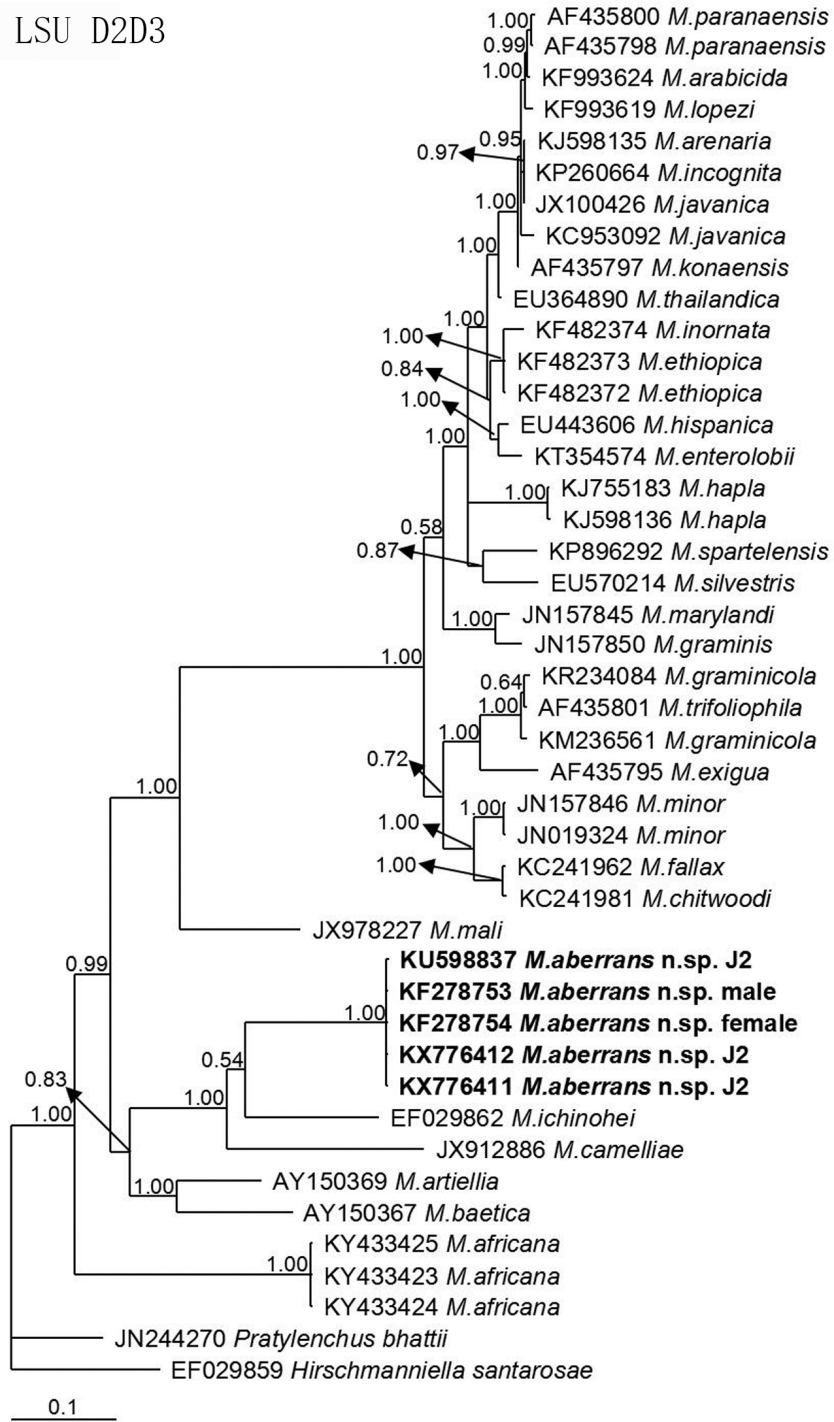
Bayesian consensus tree inferred from LSU D2D3 of *Meloidogyne aberrans* sp. nov. under GTR+G (lnL = 6787.5586; AIC = 13593.1172; freqA = 0.2278; freqC = 0.1940; freqG = 0.2868; freqT = 0.2915; R(a) = 0.8153; R(b) = 2.8655; R(c) = 1.5894; R(d) = 0.4230; R(e) = 3.6060; R(f) = 1; Pinva = 0; Shape = 0.3592). Posterior probability values exceeding 50% are given for appropriate clades.

**Fig 8:**
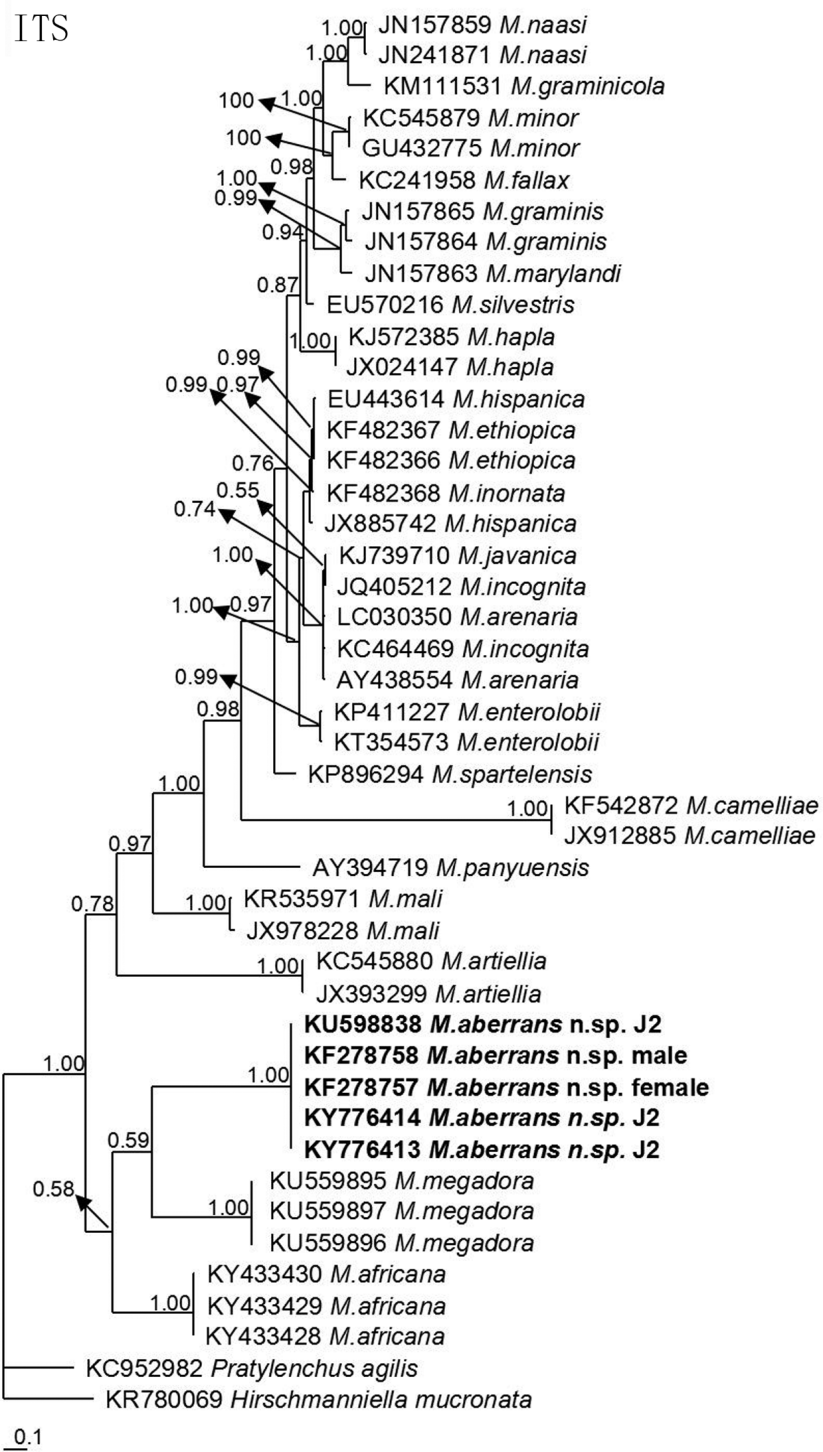
Bayesian consensus tree inferred from ITS of *Meloidogyne aberrans* sp. nov. under GTR+G+I (lnL = 10195.9648; AIC = 20411.9297; freqA = 0.2545; freqC = 0.1986; freqG = 0.2345; freqT = 0.3124; R(a) = 1.3819; R(b) = 2.3346; R(c) = 1.9930; R(d) = 0.6850; R(e) = 2.8412; R(f) = 1; Pinva = 0.0772; Shape = 1.1189). Posterior probability values exceeding 50% are given for appropriate clades.

**Fig 9:**
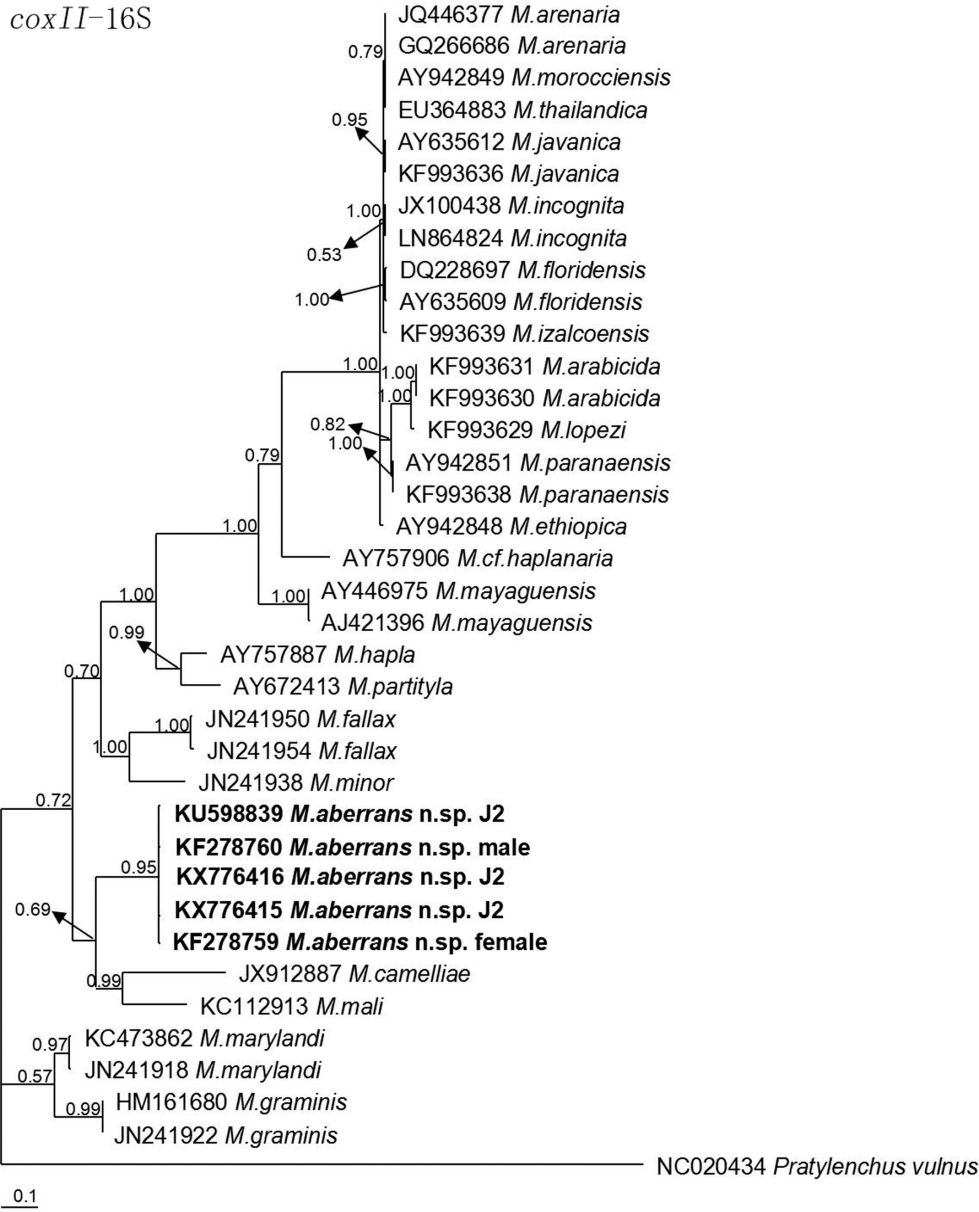
Bayesian consensus tree inferred from *coxII*-16S rRNA of *Meloidogyne aberrans* sp. nov. under TIM+G (lnL = 9069.4336; AIC = 18152.8672; freqA = 0.3360; freqC = 0.0652; freqG = 0.1289; freqT = 0.4699; R(a) = 1; R(b) = 4.2175; R(c) = 1.9168; R(d) = 1.9168; R(e) = 2.4089; R(f) = 1; Pinva = 0; Shape = 1.1069). Posterior probability values exceeding 50% are given for appropriate clades.

The molecular phylogenetic status of *M*. *aberrans* sp. nov. is presented in Figs 6–9, and based on the sequences of SSU, LSU D2D3, ITS and *coxII-*16S rRNA reconstructed in this study, these four phylogenetic trees confirmed that the new species was within the *Meloidogyne* clade. In Fig 6, the phylogenetic tree is based on SSU from a multiple alignment of 1794 total characters. When *Hirschmanniella loofi* Sher, 1968 [68] was used as the out-group taxon, *M*. *aberrans* sp. nov. was in a 100% supported monophyletic clade with *M*. *ichinohei*, another species with an elevated perineum. This clade was sister to *M*. *camelliae*, a species without an elevated perineum, but was far from the other four species that have a posterior protuberance, *M*. *graminis*, *M*. *spartinae*, *M*. *oryzae* and *M*. *africana*. In Fig 7, the phylogenetic tree is based on LSU D2D3 from a multiple alignment of 803 total characters. Using *Hirschmanniella santarosae* De Ley, Mundo ocampo, Yoder & De Ley, 2007 [69] as the out-group taxon, *M*. *aberrans* sp. nov. was also close to *M*. *ichinohei* with 54% support. These two species were also sister to *M*. *camelliae*. In Fig 8, the phylogenetic tree is based on ITS from a multiple alignment of 884 total characters. When using *Hirschmanniella mucronata* (Das, 1960) Luc & Goodey, 1963 [70,71] as the out-group taxon, *M*. *aberrans* sp. nov. and the other species *M*. *megadora* that possesses an elevated perineum were monophyletic with 59% support. This clade clustered with *M*. *africana*, a species also has an elevated perineum, with 58% support. However, the clade was far from the other species *M*. *graminis* that has a posterior protuberance. In Fig 9, the phylogenetic tree is based on *coxII-*16S rRNA from a multiple alignment of 1747 total characters. Using *Pratylenchus vulnus* Allen and Jensen, 1951 [72] as the out-group taxon, *M*. *aberrans* sp. nov. was placed in a clade with *M*. *camelliae* and *M*. *mali* with 69% support. *M*. *aberrans* sp. nov. and *M*. *graminis* (another species with an elevated perineum) were always paraphyletic in all phylogenetic trees.

### Histopathology

**Fig 10:**
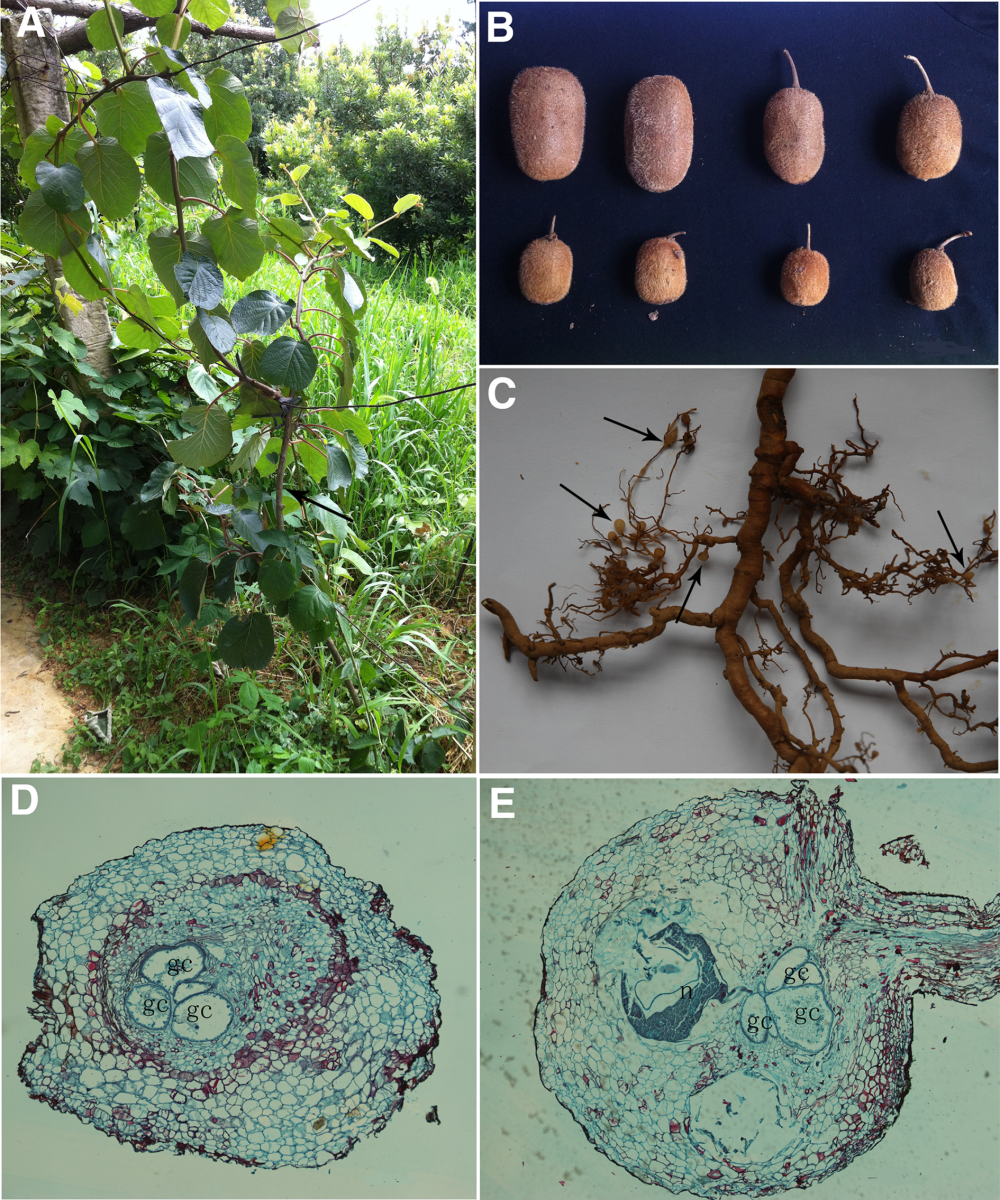
Symptoms and histopathology of wild kiwifruit infected by *Meloidogyne aberrans* sp. nov. (A) Infested tree (arrow). (B) Fruits from healthy tree (upper) and infested tree (bottom). (C) Roots with severe root galling (arrows). (D) Transverse section of root infected with *M*. *aberrans* sp. nov. (E) Longitudinal section of root infected with *M*. *aberrans* sp. nov. (gc = giant cells; n = nematode).

The wild kiwifruit infected by *M*. *aberrans* sp. nov. showed disease symptoms similar to nutritional deficiency, with dwarf plants and small sized fruits (Fig 10A and 10B). Most galls induced by *M*. *aberrans* sp. nov. on kiwifruit roots were on root tips, and the galls were oval or rounded and relatively large (approximately three- to seven-fold larger than the root diameter) (Fig 10C). Typically, a simple gall contained one to ten females that deposited an egg mass within the root tissue. Histopathological observations showed that *M*. *aberrans* sp. nov. induced formation of the large multinucleate feeding cells known as giant cells, with dense cytoplasm and thickened walls, inside the vascular cylinder. Typically, three to six giant cells were at each feeding site, which resulted in a disorganized stele (Fig 10D and 10E).

## Discussion

*Meloidogyne* is one of the most damaging plant parasites, causing approximately $70 billion in economic losses annually [73]. To date, approximately one hundred nominal species are recognized within the genus *Meloidogyne*. When based only on morphology, the identification of species in the genus *Meloidogyne* is challenging primarily because of the intraspecific variability and the interspecific overlap. Parthenogenesis and high rates of reproduction of root-knot nematodes also increase the difficulty in identification. Therefore, isozyme electrophoresis and molecular techniques greatly assist in the identification of *Meloidogyne* spp. [74]. In this study, a species of root-knot nematode that parasitizes kiwifruit in China was identified as *Meloidogyne aberrans* sp. nov., based on morphological characters, isozyme and molecular analyses.

The identification of *M*. *aberrans* sp. nov. was relatively easy because the species has a unique combination of characters that include a prominent posterior protuberance, faint perineal pattern in females, depression in outline at the oral aperture and very short, poorly defined hyaline tail terminus in J2s. Additionally, according to the rule described by Esbenshade and Triantaphyllou [29], *M*. *aberrans* sp. nov. had a rare esterase profile, S2.

Notably, females of *M*. *aberrans* sp. nov. had a prominent posterior protuberance, which is rare in the genus *Meloidogyne*. *Meloidogyne* species with an elevated perineum were previously assigned to the genus *Hypsoperine*. The genus *Hypsoperine* was proposed by Sledge and Golden in 1964 [53] for *Hypsoperine graminis* Sledge and Golden, 1964 as the type species and for *H*. *acronea* Coetzee, 1956 [50]. Sledge and Golden differentiated *Hypsoperine* from *Meloidogyne* by the prominent posterior protuberance and a thick cuticle [53], and these authors believed that the new genus *Hypsoperine* occupied a position between *Heterodera* and *Meloidogyne* that was closer to *Meloidogyne*. Subsequently, *H*. *spartinae* Rau and Fassuliotis, 1965 [60], *H*. *ottersoni* Thorne, 1969 [57], *H*. *megriensis* Poghossian, 1971 [75] and *H*. *propora* Spaull, 1977 [59] were added to the genus *Hypsoperine*. However, the taxonomic status of the genus *Hypsoperine* has always been in dispute. Whitehead (1968), Franklin (1971), Esser et al. (1976), Jepson (1987), Luc et al. (1988), Eisenback and Triantaphyllou (1991) and Araki (1992) considered *Hypsoperine* a junior synonym of *Meloidogyne* [23,53,54,58,76–78], but Golden (1971) and Handoo et al. (1993) suggested *Hypsoperine* was valid [79,80]. Siddiqi (1986) agreed with the statement of Golden (1971) [81], although he synonymized *Hypsoperine* with *Meloidogyne* in 2000 [82]. Recently, a phylogenetic tree inferred from 18S sequences placed *M*. *spartinae* (*= H*. *spartinae*) within the genus *Meloidogyne*, which showed that *Hypsoperine* should be a junior synonym of *Meloidogyne* [83]. Similarly, our phylogenetic trees also placed those *Meloidogyne* species with an elevated perineum, including *M*. *aberrans* sp. nov., *M*. *graminis*, *M*. *spartinae*, *M*. *ichinohei*, *M*. *oryzae*, *M*. *africana* and *M*. *megadora* within the *Meloidogyne* genus, although these species did not form monophyletic groups. Additionally, histopathological observations showed that *M*. *aberrans* sp. nov. induced formation of multinucleate giant cells, which was consistent with the biological characteristics of root-knot nematodes. Thus, in our study, both histopathological observations and molecular phylogenies indicate that *Hypsoperine* is a synonym of *Meloidogyne*.

Kiwifruit is widely cultivated in Guizhou, China [19], and this new species may be indigenous to Guizhou and may threaten kiwifruit in China by causing symptoms such as severe root knot and dwarfed and reduced fruit size. Additional investigations are required to determine the distribution of *M*. *aberrans* sp. nov. beyond the type locality. Moreover, further studies should be conducted to determine the host range of the new species and the optimum methods for control.

## References

[1] Zhang JY, Mo ZH, Huang SN, Guo ZR. [Development of kiwifruit industry in the world and analysis of trade and international competitiveness in China entering 21st century]. Chinese Agr. Sci. Bull. 2014; 30:48–55. Chinese.

[2] Akyazi F, Felek AF. Molecular identification of root-knot nematode *Meloidogyne incognita* from kiwi fruit orchards in Ordu province, Turkey. Turk. J. Entomol. 2013; 37:449–456.

[3] Haygood RA, Saunders JA, Miller RW. Widespread occurrence of *Meloidogyne incognita* on kiwifruit in the coastal areas of South Carolina. Plant Dis. 1990; 74:81.

[4] Khan ML. Occurrence of root-knot nematode (*Meloidogyne incognita*) and other plant parasitic species on kiwi fruit (*Actinidia delicious* Chev.) in Himachal Pradesh. Indian J. Nematol. 2000; 30:245.

[5] Philippi I, Latorre BA, Perez GF, Castillo L. Identification of the root-knot nematodes (*Meloidogyne* spp.) on kiwifruit by isoenzyme analysis in Chile. Fitopatologia 1996; 31:96–101.

[6] Somavilla L, Gomes CB, Carneiro RMDG, Carbonri J. Levantamento e caracterização de espécies do nematoide das galhas em quivi no Rio Grande do Sul. Trop. Plant Pathol. 2011; 36:89–94.

[7] Zhang SS, Gao RX. [Identification of root-knot nematode species parasitizing *Actinidia* in Fujian, China]. J. Fujian Agr. Coll. 1993; 22:433–435. Chinese.

[8] Knight KWL. Plant parasitic nematodes associated with six subtropical crops in New Zealand. New Zeal. J. Crop Hort. 2001; 29:267–275.

[9] Ma KC, Jo YS, Kim BH, Lim DG. Seasonal occurrence and aspects of root-knot nematodes in major kiwifruit cultivation areas of Korea. Acta Hortic. 2007; 753:719–724.

[10] Pinochet J, Verdejo S, Soler A. Observations on the seasonal fluctuation of *Meloidogyne hapla* on kiwi (*Actinidia deliciosa*) in Spain. Nematropica 1990; 20:31–37.

[11] Roccuzzo G, Ciancio A, Bonsignore R. Population density and soil antagonists of *Meloidogyne hapla* infecting kiwi in southern Italy. Fundam. Appl. Nematol. 1993; 16:151–154.

[12] Waliullah M. Nematodes associated with kiwi (*Actinidia deliceous* Chev.) in kashmir valley, India. Indian J. Nematol. 2005; 35:227.

[13] Carneiro RM, Randig O, Almeidav MRA, Gomes ACM. Additional information on *Meloidogyne ethiopica* Whitehead, 1968 (Tylenchida: Meloidogynidae), a root-knot nematode parasitising kiwi fruit and grape-vine from Brazil and Chile. Nematology 2004; 6:109–123.

[14] Carneiro RM, Almeida MRA, Cofcewicz ET, Magunacelaya JC, Aballay E. *Meloidogyne ethiopica*, a major root-knot nematode parasitising *Vitis vinifera* and other crops in Chile. Nematology 2007; 9:633–639.

[15] Conceição IL, Tzortzakakis EA, Gomes P, Abrantes I, Da Cunha MJ. Detection of the root-knot nematode *Meloidogyne ethiopica* in Greece. Eur. J. Plant Pathol. 2012; 134:451–457.

[16] Li SJ, Yu Z. [A new species of root-knot nematode (*Meloidogyne actinidiae*) on *Actinidia chinensis* in Henan Province]. J. Henan Agr. Univ. 1991; 25:251–253. Chinese.

[17] Somavilla L, Gomes CB, Antunes LE, de Oliveira RP, Carneiro RM. Reação de diferentes frutíferas a *Meloidogyne ethiopica*. Nematol. Bras. 2009; 33:252–255. Portuguese

[18] Yang QP, Wang LH. Xie ZB, Hu N. [The occurring regularity and ODM Technology of organic kiwifruit disease in Hubei Province]. Hubei Agr. Sci. 2014; 53:2307–2311. Chinese.

[19] Huang W, Wang CM, Qiao R. [Current situation and countermeasures of kiwifruit industry development in GuiZhou Province]. Guizhou Agr. Sci. 2012; 40:184–186. Chinese.

[20] Liang CF. [On the distribution of *Actinidias*]. Guihaia 1983; 3:229–248. Chinese.

[21] Zheng YQ, Li ZZ, Huang HW. [Preliminary study on SSR analysis in kiwifruit cultivars]. J. Wuhan Bot. Res. 2002; 21:444–448. Chinese.

[22] Li TQ, Huang YX, Zhu JX, Xia ZM. [Damage investigation and integrated control of *Meloidogyne incognita* on kiwi in Xiuwen]. Till. Cultivation. 2014; 34:68–69. Chinese.

[23] Jepson SB. Identification of root-knot nematodes (*Meloidogyne* species) Farnham Royal, UK: Commonwealth Agricultural Bureaux; 1987.

[24] Feng ZX. [Plant Nematology]. Beijing: Chinese Agricultural Publishing; 2001. Chinese.

[25] Huang G, Dong R, Allen REX, Davis EL, Baum TJ, Hussey RS. Two chorismate mutase genes from the root-knot nematode *Meloidogyne incognita*. Mol. Plant Pathol. 2005; 6:23–30. doi: 10.1111/j.1364-3703.2004.00257.x 20565635 10.1111/j.1364-3703.2004.00257.x

[26] Hartman KM, Sasser JN. Identification of *Meloidogyne* species on the basis of differential host test and perineal-pattern morphology In: Barker KR, Carter CC, Sasser JN, editors. An Advanced Treatise on *Meloidogyne*, Volume II Methodology. North Carolina: North Carolina University Graphics; 1985 p. 69–77.

[27] Eisenback JD. Techniques for preparing nematodes for scanning electron microscopy In: Barker KR, Carter CC, Sasser JN, editors. An Advanced Treatise on *Meloidogyne*, Volume II Methodology. North Carolina: North Carolina University Graphics; 1985 p. 79–105.

[28] Hu MX, Zhuo K, Liao JL. Multiplex PCR for the simultaneous identification and detection of *Meloidogyne incognita*, *M*. *enterolobii*, and *M*. *javanica* using DNA extracted directly from individual galls. Phytopathology 2011; 101:1270–1277. doi: 10.1094/PHYTO-04-11-0095 21770774 10.1094/PHYTO-04-11-0095

[29] Esbenshade PR, Triantaphyllou AC. Electrophoretic methods for the study of root-knot nematode enzymes In: Barker KR, Carter CC, Sasser JN, editors. An Advanced Treatise on *Meloidogyne*, Volume II Methodology. North Carolina: North Carolina University Graphics; 1985 p. 79–105.

[30] Mundo-Ocampo M, Troccoli A, Subbotin SA, Del Cid J, Baldwin JG, Inserra RN. Synonymy of *Afenestrata* with *Heterodera* supported by phylogenetics with molecular and morphological characterisation of *H*. *koreana* comb. n. and *H*. *orientalis* comb. N. (Tylenchida: Heteroderidae). Nematology 2008; 10:611–632.

[31] Holterman M, van der Wurff A, van den Elsen S, van Megen H, Bongers T, Holovachov O, et al Phylum-wide analysis of SSU rDNA reveals deep phylogenetic relationships among nematodes and accelerated evolution toward crown clades. Mol. Biol. Evol. 2006; 23:1792–1800. doi: 10.1093/molbev/msl044 16790472 10.1093/molbev/msl044

[32] Subbotin SA, Sturhan D, Chizhov VN, Vovlas N, Baldwin JG. Phylogenetic analysis of Tylenchida Thorne, 1949 as inferred from D2 and D3 expansion fragments of the 28S rRNA gene sequences. Nematology 2006; 8:455–474.

[33] Subbotin SA, Waeyenberge L, Moens M. Identification of cyst forming nematodes of the genus *Heterodera* (Nematoda: Heteroderidae) based on the ribosomal DNA RFLPs. Nematology 2000; 2:153–164.

[34] Powers TO, Harris TS. A polymerase chain reaction method for identification of five major *Meloidogyne* species. J. Nematol. 1993; 25:1–6. 19279734 PMC2619349

[35] Tanha Maafi Z, Subbotin SA, Moens M. Molecular identification of cyst-forming nematodes (Heteroderidae) from Iran and a phylogeny based on the ITS sequences of rDNA. Nematology 2003; 5:99–111.

[36] Zhuo K, Cui RQ, Ye WM, Luo M, Wang HH, Hu XN, Liao JL. Morphological and molecular characterization of *Aphelenchoides fujianensis* n.sp. (Nematoda: Aphelenchoididae) from *Pinus massonianain* China. Zootaxa 2010; 2509:39–52.

[37] Castillo P, Vovlas N, Subbotin S, Troccoli A. A new root-knot nematode, *Meloidogyne baetica* n.sp. (Nematoda: Heteroderidae), parasitizing wild olive in Southern Spain. Phytopathology 2003; 93:1093–1102. doi: 10.1094/PHYTO.2003.93.9.1093 18944092 10.1094/PHYTO.2003.93.9.1093

[38] Castillo P, Vovlas N, Troccoli A, Liébanas G, Palomares Rius JE, Landa BB. A new root-knot nematode, *Meloidogyne silvestris* n. sp. (Nematoda: Meloidogynidae), parasitizing European holly in northern Spain. Plant Pathol. 2009; 58:606–619.

[39] Kiewnick S, Holterman M, van den Elsen S, van Megen H, Frey JE, Helder J. Comparison of two short DNA barcoding loci (*COI* and *COII*) and two longer ribosomal DNA genes (SSU & LSU rRNA) for specimen identification among quarantine root-knot nematodes (*Meloidogyne* spp.) and their close relatives. Eur. J. Plant Pathol. 2014; 140:97–110.

[40] Tamura K, Dudley J, Nei M, Kumar S. MEGA4: molecular evolutionary genetics analysis (MEGA) software version 4.0. Mol. Biol. Evol. 2003; 24:1596–1599. 10.1093/molbev/msm092 17488738

[41] Posada D, Crandall KA. Modeltest: testing the model of DNA substitution. Bioinformatics 1998; 14:817–818. 9918953 10.1093/bioinformatics/14.9.817

[42] Huelsenbeck JP, Ronquist F. MRBAYES: Bayesian inference of phylogenetic trees. Bioinformatics 2001; 17:1754–1755. 10.1093/bioinformatics/17.8.754 11524383

[43] Swofford DL. PAUP*-Phylogenetic Analyses Using Parsimony (and other Methods). Version 4 b10. Sunderland; Sinauer Associates; 1998.

[44] Larget B, Simon DL. Markov chain Monte Carlo algorithms for the Bayesian analysis of phylogenetic trees. Mol. Biol. Evol. 1999; 16:750–759.

[45] Tepper HB. Benzyladenine promotes shoot initiation in empty leaf axils of Stellaria media l. J. Plant Physiol. 1992; 140:241–243.

[46] Tepper HB. Developmental features accompanying the imposition and release of apical dominance in pea. J. Plant Physiol. 1993; 142:722–729.

[47] Bachand GD, Castello JD. Immunolocalization of tomato mosaic tobamovirus in roots of red spruce seedlings. J. Phytopathol. 2001; 149:415–419.

[48] Castillo P, Vovlas N, Jiménez-Díaz RM. Pathogenicity and histopathology of *Pratylenchus thornei* populations on selected chickpea genotypes. Plant Pathol. 1998; 47:370–376.

[49] Araki M. Description of *Meloidogyne ichinohei* n. sp. (Nematoda: Meloidogynidae) from Iris laevigata in Japan. Japan. J. Nematol. 1992; 22:11–20.

[50] Coetzee V. *Meloidogyne acronea*, a new species of root-knot nematode. Nature 1956; 4515:899–900. 10.1038/177899a0 13321999

[51] Whitehead AG. The root-knot nematodes of east Africa *Meloidogyne africana* n. sp., a parasite of arabica coffee (*Coffea arabica* L.). Nematologica. 1959(4):272–8.

[52] Janssen T, Karssen G, Topalović O, Coyne D, Bert W. Integrative taxonomy of root-knot nematodes reveals multiple independent origins of mitotic parthenogenesis. Plos One. 2017(3): e0172190 doi: 10.1371/journal.pone.0172190 28257464 10.1371/journal.pone.0172190 PMC5336219

[53] Sledge EB, Golden AM. *Hypsoperine graminis* (Nematoda: Heteroderidae), a new genus and species of plant-parasitic nematode. P. Helminthol. Soc. Wash. 1964; 31:83–88.

[54] Whitehead AG. Taxonomy of *Meloidogyne* (Nematodea: Heteroderidae) with descriptions of four new species. Trans. Zool. Soc. London 1968; 31:263–401.

[55] Maleita CM, Almeida AF, Vovlas N, Abrantes I. Morphological, Biometrical, Biochemical, and Molecular Characterization of the Coffee Root-Knot Nematode Meloidogyne megadora. Plant Disease. 2016(8): 1725–1734. 10.1094/PDIS-01-16-0112-RE 30686217

[56] Siddiqi MR, Booth W. *Meloidogyne (Hypsoperine) mersa* sp. n. (Nematoda: Tylenchina) attacking *Sonneratia alba* trees in mangrove forest in Brunei Darussalam. Afro-Asian J. Nematol. 1991; 1:212–220.

[57] Thorne G. *Hypsoperine ottersoni* sp. n. (Nemata, Heteroderidae) infesting canary grass, *Phalaris arundinacea* (L.) reed in Wisconsin. P. Helminthol. Soc. Wash. 1969; 36:98–102.

[58] Franklin MT. Taxonomy of Heteroderidae In: Zuckerman BM, Mai WF, Rohde RA, editors. Plant Parasitic Nematodes. Volume 1. Morphology, Anatomy, Taxonomy and Ecology. New York and London: Academic Press Inc; 1971 p. 139–162.

[59] Spaull VW. *Meloidogyne propora* n. sp. (Nematoda: Meloidogynidae) from Aldabra Atoll, Western Indian Ocean, with a note on *M*. *javanica* (Treub). Nematologica 1977; 23:177–186.

[60] Rau GJ, Fassuliotis G. *Hypsoperìne spartinae* n. sp., a gall-forming nematode on the roots of smooth cordgrass. P. Helminthol. Soc. Wash. 1965; 32:159–162.

[61] Maas PT, Sanders H, Dede J. *Meloidogyne oryzae* n. sp. (Nematoda, Meloidogynidae) infesting irrigated rice in Surinam (South America). Nematologica 1978; 24:305–311.

[62] López CR. *Meloidogyne salasi* sp. n.(Nematoda: Meloidogynidae), a new parasite of rice (Oryza sativa L.) from Costa Rica and Panama. Turrialba 1984; 34:275–286.

[63] Gaur HS, Saha M, Khan E. *Meloidogyne triticoryzae* sp. n.(Nematoda: Meloidogynidae) a root-knot nematode damaging wheat and rice in India. Annals of Plant Protection Sciences 1993; 1:18–26.

[64] Neal JC. Root-knot disease of the peach, orange, and other plants in Florida, due to the work of Anguillula Bulletin 20, Division of Entomology, US Department of Agricultural; 1889.

[65] Chitwood BG. Root-knot nematodes, part I. A revision of the genus *Meloidogyne* Goeldi, 1887. P. Helminthol. Soc. Wash. 1949; 16:90–104.

[66] Kofoid CA, White AW. A new nematode infection of man. J. Am. Med. Assoc. 1919; 72:567–569.

[67] Treub M. Onderzoekingen over sereh-ziek suikerriet gedaan in's Lands Plantentuin te Buitenzorg. Mededeelingen uit's Lands Plantentium, Btavia. 1885; 2:1–39.

[68] Sher SA. Revision of the genus Hirschmanniella Luc & Goodey, 1963 (Nematoda: Tylenchoidea). Nematologica 1968; 14:243–275.

[69] De Ley I, Mundo ocampo M, Yoder M, De Ley P. Nematodes from vernal pools in the Santa Rosa Plateau Ecological Reserve, California I. *Hirschmanniella santarosae* sp. n. (Nematoda: Pratylenchidae), a cryptic sibling species of *H*. *pomponiensis* Abdel-Rahman & Maggenti, 1987. Nematology 2007; 9:405–429.

[70] Das VM. Studies on the nematode parasites of plants in Hyderabad (Andhra Pradesh, India). Z. Parasitenkd. 1960; 19:553–605. 13814174 10.1007/BF00260158

[71] Luc M, Goodey JB. *Hirschmanniella* nom. nov. for *Hirschmannia*. Nematologica 1963; 9:471.

[72] Allen MW, Jensen HJ. *Pratylenchus vulnus*, new species (Nematoda Pratylenchinae), a parasite of trees and vines in California. P. Helminthol. Soc. Wash. 1951; 18:47–50.

[73] Caboni P, Ntalli NG, Aissani N, Cavoski I, Angioni A. Nematicidal activity of (E, E)-2,4-decadienal and (E)-2-decenal from Ailanthus altissima against *Meloidogyne javanica*. J. Agr Food Chem. 2012; 60:1146–1151. 22224661 10.1021/jf2044586

[74] Hunt DJ, Handoo ZA. Taxonomy, identification and principal species In: Moens M, Perry RN, Starr JL, editors. Root-knot nematodes. Wallingford, UK: CABI 2009 p. 55–97.

[75] Poghossian EE. [*Hypsoperine megriensis* n. sp. (Nematoda: Heteroderidae) in the Armenian SSR, a gall forming nematode on the roots of smooth cordgrass]. Dokl. Akad. Nauk Armyanskoi SSR 1971; 53:306–312. Russian.

[76] Esser RP, Perry VG, Taylor AL. A diagnostic compendium of the genus *Meloidogyne* (Nematoda: Heteroderidae). P. Helminthol. Soc. Wash. 1976; 43:138–150.

[77] Luc M, Maggenti AR, Fortuner R. A reappraisal of Tylenchina (Nemata). 9. The family Heteroderidae Filip’ev and Schuurmans Stekhoven, 1941. Rev. Nématol. 1988; 11:159–176.

[78] Eisenback JD, Triantaphyllou HH. Root-knot nematodes: *Meloidogyne* species and races In: Nickle WR, editor. Manual of Agricultural Nematology. New York: Marcel Dekker, INC; 1991 p. 191–274.

[79] Golden AM. Classification of the genera and higher categories of the order Tylenchida (Nematoda) In: Zuckerman BM, Mai WF, Rohde RA, editors. Plant Parasitic Nematodes. Volume I. Morphology, Anatomy, Taxonomy and Ecology. New York and London: Academic Press Inc; 1971 p. 191–232.

[80] Handoo ZA, Huettel RN, Golden AM. Description and SEM observations of *Meloidogyne sasseri* n. sp. (Nematoda, Meloidogynidae), parasitizing beachgrasses. J. Nematol. 1993; 25:628–641. 19279820 PMC2619418

[81] Siddiqi MR. Tylenchida: parasites of plants and insects Farnham Royal, UK: Commonwealth Agricultural Bureaux; 1986.

[82] Siddiqi MR. Tylenchida: parasites of plants and insects, 2nd edn. Wallingford, UK: CABI; 2000.

[83] Plantard O, Valette S, Gross MF. The root-knot nematode producing galls on *Spartina alterniflora* belongs to the genus *Meloidogyne*: Rejection of *Hypsoperine* and *Spartonema* spp. J. Nematol. 2007; 39:127–132. 19259481 PMC2586490

